# Effect of nonsteroidal anti-inflammatory drugs (NSAIDs) association on physicochemical and biological properties of tricalcium silicate-based cement

**DOI:** 10.1590/0103-6440202204644

**Published:** 2022-06-24

**Authors:** Maria Carolina Guiotti de Oliveira, Índia Olinta de Azevedo Queiroz, Thiago Machado, Lorena de Mello Alcântara Garrido, Sandra Helena Penha de Oliveira, Marco Antonio Hungaro Duarte

**Affiliations:** 1 Department of Dentistry, Endodontics and Dental Materials, Bauru School of Dentistry, USP, Bauru, São Paulo, Brazil; 2 Department of Oral and Maxillofacial Surgery and Integrated Clinic, Araçatuba Dental School, UNESP, Araçatuba, São Paulo, Brazil; 3 Department of Basic Science, Araçatuba Dental School, UNESP, Araçatuba, São Paulo, Brazil

**Keywords:** Nonsteroidal anti-inflammatory, endodontic, diclofenac sodium, tricalcium silicate

## Abstract

The aim of this study was to investigate the physicochemical and biological properties of an experimental tricalcium silicate-based repair cement containing diclofenac sodium (CERD). For the physicochemical test, MTA, Biodentine and CERD were mixed and cement disc were prepared to evaluate the setting time and radiopacity. Root-end cavity were performed in acrylic teeth and filled with cements to analyze the solubility up to 7 days. Polyethylene tubes containing cements were prepared and calcium ions and pH were measured at 3h, 24h, 72h and 15 days. For the biological test, SAOS-2 were cultivated, exposed to cements extracts and cell proliferation were investigated by MTT assay at 6h, 24h and 48h. Polyethylene tubes containing cements were implanted into *Wistar* rats. After 7 and 30 days, the tubes were removed and processed for histological analyses. Parametric and nonparametric data were performed. No difference was identified in relation to setting time, radiopacity and solubility. Biodentine released more calcium ion than MTA and CERD; however, no difference between MTA and CERD were detected. Alkaline pH was observed for all cements and Biodentine exhibited highest pH. All cements promoted a raise on cell proliferation at 24h and 48h, except CERD at 48h. Biodentine stimulated cell metabolism in relation to MTA and CERD while CERD was more cytotoxic than MTA at 48h. Besides, no difference on both inflammatory response and mineralization ability for all cement were found. CERD demonstrated similar proprieties to others endodontic cements available.

## Resumo

O objetivo deste estudo foi investigar as propriedades físico-químicas e biológicas de um cimento reparador experimental à base de silicato de tricálcio contendo diclofenaco de sódio (CERD). Para o teste físico-químico, MTA, Biodentine e CERD foram manipulados e discos de cimentos foram preparados para avaliar o tempo de presa e a radiopacidade. Retrocavidades foram feitas em dentes de acrílico e preenchidas com cimentos para análise de solubilidade por 7 dias. Tubos de polietileno contendo cimentos foram preparados e os íons cálcio e o pH foram mensurados às 3h, 24h, 72h e 15 dias. Para o teste biológico, SAOS-2 foram cultivadas, expostas aos extratos de cimentos e a proliferação celular foi investigada pelo ensaio de MTT às 6h, 24h e 48h. Tubos de polietileno contendo cimentos foram implantados em ratos *Wistar*. Após 7 e 30 dias, os tubos foram removidos e processados para análises histológicas. Dados paramétricos e não paramétricos foram realizados. Nenhuma diferença foi identificada em relação ao tempo de presa, radiopacidade e solubilidade. Biodentine liberou mais íons de cálcio do que MTA e CERD; no entanto, nenhuma diferença entre MTA e CERD foi detectada. O pH alcalino foi observado para todos os cimentos e o Biodentine exibiu o pH mais alto. Todos os cimentos promoveram aumento na proliferação celular às 24h e 48h, exceto o CERD às 48h. Biodentine estimulou o metabolismo celular em relação ao MTA e CERD, enquanto CERD foi mais citotóxico do que MTA em 48h. Além disso, nenhuma diferença foi encontrada na resposta inflamatória e na capacidade de mineralização para todos os cimentos. CERD demonstrou propriedades semelhantes a outros cimentos endodônticos disponíveis.

## Introduction

Tricalcium silicate-based cements were developed over a decade ago and are widely used in several endodontic procedures related to pulp and periapical tissues, such as pulp cap, pulpotomy, perforation, filling and root-end filling ^1^. Mineral Trioxide Aggregate (MTA) was the first tricalcium silicate-based cement developed and it is composed of fine hydrophilic particles of tricalcium silicate, dicalcium silicate, tricalcium aluminate, tricalcium oxide, silicate oxide and bismuth oxide as a radiopacifier ^1^ ^,^ ^2^. Biodentine is a repair cement based on synthetic tricalcium silicate which was designed as a dentine substitute material and similar to MTA it is also used in pulpotomies, perforations and as a root-end filling ^2^. Investigations showed that these both cements are biocompatible, capable of induce differentiation of dental pulp stem cells and promotes hard tissues repair ^3^ ^,^ ^4^. 

It is already described that tricalcium silicate-based cement exhibit antibacterial activity and induce intense inﬂammatory reaction at initial times; however, these both properties are dependent of cement ability to release hydroxyl ions and raise pH values ^5^. Due this, some substances that enhance antibacterial activity and decrease the inflammatory process were sought out, including the nonsteroidal anti-inflammatory drugs (NSAIDs). These drugs commonly used to relieve pain and inflammation, acting on COX enzyme and inhibiting the synthesis of its metabolites, such as PGE_2_ ^6^; however, these drugs can have an adverse effect on bone tissue, impairing the growth of osteoblasts ^7^. 

Diclofenac sodium is NSAIDs that has already been studied in Endodontics for postoperative pain ^8^ or associated with calcium hydroxide paste ^9^ ^,^ ^10^ and with tricalcium silicate-based cements ^11^, as it has a highly bactericidal action ^12^. So, due to beneficial effects/properties of this drug and the lack in the literature regarding the use of NSAIDs, especially the diclofenac sodium, as one of the components of endodontic cement, which could be extremely innovative alternative in Endodontics. The aim of this study was to evaluate the physicochemical and biological properties of an experimental tricalcium silicate-based repair cement that contains diclofenac sodium (CERD) looking for their development and commercialization. The null hypothesis tested is that the CERD presents similar properties than the others endodontic cements available, MTA and Biodentine. 

## Material and Methods

An a priori sample size calculation was performed using G*Power software version 3.1 for Mac (Heinrich-Heine, Universität Düsseldorf, Düsseldorf, NRW, Germany). For all variable evaluated ANOVA test was used with an alpha error probability of 0.05 and a power of 90%. The effect size was based on previous studies with similar methodology, being 12.72 for setting time and 1.15 for radiopacity, therefore, a sample size of 2 specimens was recommended for both. For pH levels, calcium ions release, and solubility were found 1.039, 0.711 and 1.731 as the effect size for each variable respectively and 10 specimen per group was indicated as the ideal sample size. Samples that presented microcracks, cracks and voids were excluded from the study and all analyses were performed by blinded examiners.

MTA Angelus (Angelus Indústria de Produtos Odontológicos S/A, Londrina, Brazil) mixed using 1g of powder to 0.3mL liquid proportion (MTA/distilled water); Biodentine (Septodont, Saint Maur des Fausse´s, France) mixed using 1g of powder to 0.25mL liquid proportion (Biodentine/liquid) and following manufacturer’s recommendations and CERD mixed in the ratio of 1g powder: 0.4mL (CERD/vehicle) were used in this study (Table 1). In addition, American National Standards Institute (ANSI)/ADA N^o^. 57 and ISO 6876 specifications were followed for the setting time, radiopacity and solubility test.

**Table 1:** Composition of the cements

Materials	Composition/Proportion
MTA Angelus	Powder: silicon oxide, potassium oxide, aluminum oxide, sodium oxide, iron oxide, calcium oxide, bismuth oxide, magnesium oxide, insoluble residues and crystalline silica.
	Liquid: distilled water.
	Proportion: 1g of powder to 0.3mL liquid
Biodentine	Powder: tricalcium silicate, dicalcium silicate, calcium carbonate, calcium oxide and zirconium oxide.
	Liquid: water, calcium chloride, hydrosoluble polymer.
	Proportion: 1g powder to 0.25mL liquid
CERD	Powder: tricalcium silicate, zirconium oxide, calcium phosphate, calcium tungstate and diclofenac sodium.
	Liquid: propylene glycol and water.
	Proportion: 1g powder to 0.4mL liquid

###  Physicochemical test 

### Setting time

The cements were mixed and placed in nine metallic rings (n = 3) (10 mm diameter and 2 mm thickness) and kept in an oven (37^o^C and 95% humidity) throughout the analysis. After 180 ± 5 seconds from the start of the spatulation, the specimens were marked with vertical pressure, first with a 113.5g Gilmore needle to determine the initial setting time, then, with a 456.3g Gilmore needle for the final setting time. The times were registered in minutes with a digital chronometer. 

### Radiopacity

For the radiopacity test, the cements were mixed and three cylindrical samples (10 mm diameter and 1mm height) were prepared and stored in an oven at 37^o^C for setting ^13^. After, the specimens were radiographed on Kodak occlusal radiographic films (Kodak Comp, Rochester, NY, USA) using a radiographic unit (Gnatus XR 6010, Gnatus, Ribeirão Preto, SP, Brazil). Then, the radiographic were processed, digitized and radiographic density values were evaluated and converted into aluminum (mm Al) according to the formula proposed by Húngaro ^14^. 

### pH levels and calcium ions release

The pH level measurement was performed with a digital pHmeter (model 371; Micronal, São Paulo, SP, Brazil) previously calibrated. Ten polyethylene tubes (10mm length and 1mm diameter) were filled with the cements. The specimens were individually placed in test tubes containing 10 mL of deionized water and kept in an incubated at 37^o^C. The tube was sealed in a flask containing 10 mL of deionized water. The pH measurements were taken at 3h, 24h, 72h and 15 days. After, these measurements, the amount of calcium released into the deionized water was determined at 3h, 24h, 72h and 15 days using an inductively coupled plasma optical emission spectrometer - ICP-OES Optima 8300 series (PerkinElmer, São Paulo, SP, Brazil).

### Solubility Analysis

Root-end cavities (diameter: 0.5mm; height: 3mm) were performed following early studies ^13^ ^,^ ^15^ in thirty acrylic teeth (n=10) and cements filled. Immediately after filling, the specimens were scanned with using a desktop x-ray micro-focus computed tomographic scanner (SkyScan 1174v2; SkyScan, Kontich, Belgium). The scanning procedure parameters were as follows: 50 kV x-ray tube voltages, 800 mA anode current, voxel size of 14.01μm, 0.8° rotation steps, and a 360° rotation Subsequently, the specimens were immersed in plastic bottles containing 15 mL of 15 mL ultrapure water and stored at 37°C and 100% humidity for 07 days. Then, the specimens were removed from their bottles and new scanning was performed using the same parameters used in the first stage. All images were reconstructed in software (NRecon v.1.6.3; Bruker-microCT) and CTan software (CTanv1.11.10.0, SkyScan) was used to measure the sample volume (mm^3^). The solubility was determined by calculating by the difference in between the test specimen before and after immersion in ultrapure water and the solubility percentage was calculated by dividing the volume lost by the total volume.

###  Biological test 

### In vitro assay - Cell proliferation

Human osteosarcoma Saos-2 cells (HTB-85, American Type Culture Collection, Manassas, Va, USA) were cultured under standard cell culture conditions and cell proliferation were evaluated using MTT assay. MTA Angelus, Biodentine and CER were mixed and fresh extract were prepared. Serial extracts dilutions (undiluted, ^1^/_2_, ^1^/_4_, ^1^/_8_, ^1^/_16_, ^1^/_32_) were used. 

Briefly, Saos-2 cells were seeded in 96-well plate at (10^4^cells/well) and incubated for 24 hours to attachment the cells before addition of extracts. Then, cultures were exposed to serial extracts dilution. Saos-2 cells cultured without extract were used as control. At 6, 24, and 48hs, the cell proliferation was examined. Each condition was analyzed in triplicate. 

### In vivo assay - histological analyses

All experiments were approved by the guidelines of the Animal Ethics Committee (protocol number 00357-2017) and ARRIVE guidelines. Twelve *Wistar* male albino rats, aged 3 to 4 months, weighing 250-280g were used in the study. 

The cements were mixed, inserted into sterile polyethylene tubes (Abbott Labs of Brazil, São Paulo, Brazil) and implanted in the dorsal connective tissue of Wistar rats. Empty tubes were used as controls. After, 07 and 30 days of implantation, six animals of each group were euthanized and the implanted tubes with the surrounding tissues were removed and processed for histological analyses. The tissues were sliced into 5μm cuts and stained with hematoxylin-eosin staining. The 10μm cuts were stained using the von Kossa technique. Inflammatory reactions in the tissues close to the material were evaluated according to ISO/TR 7405-1997 as: 0, no or few inflammatory cells and no reaction; 1, fewer than 25 cells and light reaction; 2, between 25-125 cells and moderate reaction; and 3, 125 or more cells and severe reaction. 

### Statistical analysis

Previous the statistical analysis, all data obtained by means of different evaluations were submitted to D’Agostino and Pearson normality test to verify the normal distributions. Radiopacity and pH level were analysis by ANOVA followed by Tukey’s tests. Cell proliferation were analysis by Two-way ANOVA and Bonferroni tests. The calcium ion release and histologic data did not show normal distributions and were evaluated using Kruskal-Wallis test followed by Dunn multiple comparison tests. Graph Pad Prism (version 9.0) software program was used for statistical analysis. The p value were considered significant at 5%. 

## Results

### Effects of the Nonsteroidal Anti-inflammatory associations on physicochemical properties

CERD presented the highest setting time while MTA the shortest. All cements exhibited radiopacity higher than 3mmAl and no difference between them were identified (p>0.05) (Table 2). 

**Table 2:** Mean and standard deviation of the radiopacity (mm Al), setting time (min), solubility, calcium ions and pH values for the cements.

Groups	Radiopacity (mmAl)	Setting time (min)		Solubility	
		Initial	Final	Initial	Final
MTA	8.74 (0.44)^a^	10.45 ± 3.03	18.50 ± 1.51	6.07 ± 1.40^a^	5.13 ± 1.7^b^
Biodentine	8.27(1.05)^a^	1.30 ± 0.0	4.27 ± 0.66	7.53 ± 1.00^a^	5.80 ± 1.24^b^
CERD	8.84(0.47)^a^	7.15 ± 3.22	14.13 ±14.02	10.09 ± 1.06^a^	8.44 ± 1.68^b^
Calcium íons					
Groups	3h	24h	72h	15 days
MTA	8.54 ± 2.25	8.96 ± 2.46	5.54 ± 5.39	24.15 ±9.49^AB^
Biodentine	41.91 ± 25.95^a^	39.03 ± 12,65^a^	19.35 ± 6.22^ABa^	31.03 ± 13.03
CERD	19.12 ± 9.16	6.92 ± 3.33^b^	2.69 ± 3.18^Ab^	10.25 ± 4.72^Cb^
pH values				
Groups	3h	24h	72h	15 days
MTA	8.85 ± 0.39	8.59 ± 0.33	8.42 ± 0.63	9.53 ± 0.72
Biodentine	9.22 ± 0,33	9.23 ± 0.35^a^	9.48 ± 0,60^a^	10.18 ± 0.28
CERD	8.97 ± 0.24	8.49 ± 0.58^b^	8.57 ± 0,57^b^	8.93 ± 0.69ᵇ

Capital letters indicate differences intragroup observed in the comparison the same cement at different times: A: versus 3h; B: versus 24h; C: versus 15 days. Lower letters indicate differences intergroup observed in the comparison of different cement at the same time: a: versus MTA and b: versus Biodentine.

Biodentine released more calcium ions than MTA and CERD (p<0.05); however, no difference between MTA and CERD in all time were observed (p>0.05) (Table 2). Alkaline pH were observed for all cements in all times (pH above 8.0) and Biodentine was the most alkaline cement (Table 2).

### Effects of the Nonsteroidal Anti-inflammatory association on cell proliferation, tissue response and mineralization ability

**Figure 1:**
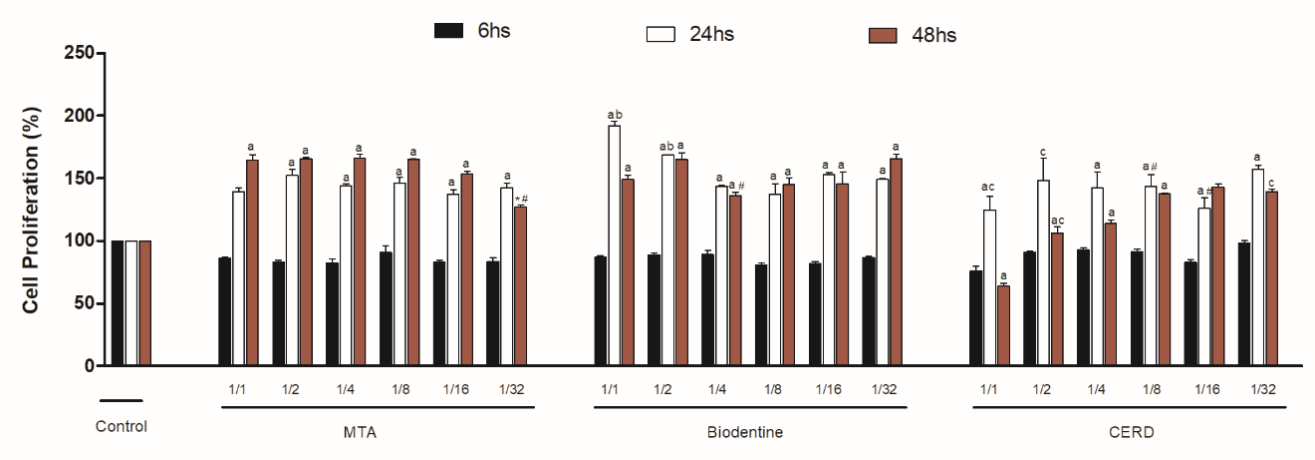
Cell proliferation observed after stimulation with diluted cement extracts at 6, 24 and 48h. The letters indicate statistical difference when comparing different association at the same dilution. (a): p<0.05 versus Control; (b): p<0.05 versus MTA; (c): p<0.05 versus Biodentine. The symbols indicate statistical difference observed comparing different extract dilution of the same material: *: p<0.05 vs. undiluted extract; #: p<0.05 vs. ½ dilution; 0: vs. ¼ dilution.

Irrespective of extract dilution the cell exposure to MTA, Biodentine and CERD promoted a raise on cell metabolism when compared with Control at 24h and 48h (p<0.05), except for CERD undiluted that reduced at 48h (p<0.05) (Figure 1). 

Regardless of the dilution used, no significant difference was observed in the presence of Biodentine (p>0.05); moreover, MTA (undiluted, ½ and ¼) promoted greater cell proliferation compared to MTA ^1^/_32_ at 48h (p<0.05). On the other hand, at 48h, CERD undiluted reduced cell proliferation when compared to CERD (^1^/_8_ and ^1^/_16_) and CER ½ decreased to CERD (^1^/_16_ and ^1^/_32_) (p<0.05) (Figure 1). Comparison between extract at the same dilution (undiluted and ½) showed that Biodentine stimulated cell metabolism in relation to MTA at 24h and CERD at 48h (p<0.05). In addition, MTA raised cellular metabolism when compared to CERD at 48h (p<0.05) (Figure 1).

A moderate inflammatory response which decreased by time (p<0.05) and mineralization areas were detected in the presence of all cements (Table 3).

**Table 3:** Inflammatory scores specimens stained with hematoxylin-eosin, thickness of fibrous capsule and biomineralization ability of all groups.

Time	Groups	Inflammatory response				Capsule	Biomineralization ability (%)
		Scores					
		0	1	2	3		
7days	Control	0/6	1/6	5/6	0/6	Thick	0
	MTA	0/6	0/6	5/6	1/6	Thick	Presence
	Biodentine	0/6	0/6	6/6	0/6	Thick	Presence
	CERD	0/6	0/6	5/6	1/6	Thick	Presence
30 days	Control	0/6	6/6	0/6	0/6	Thin	0
	MTA	0/6	5/6	1/6	0/6	Thin	Presence
	Biodentine	0/6	4/6	2/6	0/6	Thin	Presence
	CERD	0/6	4/6	2/6	0/6	Thin	Presence

Scores: 0: no or few inflammatory cells and no reaction; 1: fewer than 25 cells and light reaction; 2: between 25-125 cells and moderate reaction; 3: 125 or more cells and severe reaction.

## Discussion

Early investigations have already documented the use of diclofenac sodium in association with calcium hydroxide paste as alternative to intracanal medication ^9^ ^,^ ^10^. Moreover, any data are available concerning the use of NSAIDs in the development of cement. Despite that Ruiz-Linares et al. ^11^ evaluated the antimicrobial activity of the association between Biodentine with diclofenac sodium, to our knowledge, this is the first study that assess the use NSAIDs as an active compound of a calcium silicate-based cement and investigated their physicochemical and biologic properties. The null hypothesis of this study was rejected because differences were observed in setting time, calcium ion release, pH values and cell proliferation between the cements. 

The setting time found for Biodentine and MTA agrees with previous investigations ^16^ while CERD exhibited the highest setting time. It is important to highlight that this cement use propylene glycol as vehicle as in previous research ^17^ ^,^ ^18^ ^,^ ^19^. The hydration reaction is an important factor for the setting reaction of tricalcium silicate-based cement and the addition propylene glycol reduces the amount of water available for this reaction resulting in longer initial setting time ^17^ ^,^ ^18^ ^,^ ^19^. So, our data agree with reports that showed that addition of propylene glycol to tricalcium silicate based reparative cement increases setting time ^17^ ^,^ ^18^ ^,^ ^19^. 

All cements had radiopacity values above those recommended by ISO 6876 and our data were similar to those investigations that showed high radiopacity for MTA and Biodentine ^2^. CERD shows calcium tungstate in their composition that can improve their radiopacity ^20^ and justify our findings.

MTA and Biodentine solubility were similar to previously reports ^20^. On the other hand, Marciano et al. ^20^ demonstrated a reduction on MTA solubility after their association with 20% of propylene glycol. So, since the reduction of the amount of water available for the hydration reaction provides by the addition of propylene glycol promotes an increase on setting times ^17^ ^,^ ^18^ ^,^ ^19^ and once the solubility can be related with setting time, radiopacifier and vehicle used ^19^ ^,^ ^20^ variations in proportions/ratio of this compound in the cement composition can resulting in higher solubility, which compromises the sealing ability of the material; moreover, our data demonstrated that CERD solubility was not altered by any of them.

Biodentine was the material that most release calcium at initial time fact that can be explained due calcium chloride ^2^ ^,^ ^21^. CERD was able to release calcium ions and had a similar release rate to MTA. Natu et al. ^18^ reported an increase on calcium ion release over time in the presence of 20% of propylene glycol. However, Duarte et al. ^17^ demonstrated that of amount of calcium ion released were directly proportional of percentage of propylene glycol added. Despite that type and concentration of vehicle affects the diffusion/dissociation ability ^22^ we considered that the percentage of propylene glycol and water present in the CERD can justify our findings. By the way, any data concerning the calcium ion released from use of NSAIDs as cement compound or even from the association between NSAIDs and calcium hydroxide paste is available. 

All cements showed alkaline pH levels and these findings corroborate with early studies that evaluated the release of hydroxyl ions from MTA and Biodentine and found values greater than 8.0 ^16^. De Freitas et al. ^9^ evaluated the pH values of calcium hydroxide paste associated with different NSAIDs and showed that the lowest values were found in those that containing diclofenac sodium; however, in all periods, the pH remained above 10 showing that the association did not interfere in the alkalinity. In addition, some investigations related that the addition of propylene glycol to MTA did not interfere in alkalinity ^9^ ^,^ ^17^. Therewith, since the ionic dissociation are influenced by the chemical composition of cement, we considered that the presence of both propylene glycol and diclofenac sodium in the CERD composition could explain ours results. 

Although of physicochemical studies are widely used to investigate if the material shows adequate propriety and also handling characteristics to be used as a biomaterial, any material can be applied in clinical without completing a biological examination and evaluation of host-biomaterial interaction. Thus, to better understanding the effects of NSAIDs as endodontic cement a cell proliferation assay and biocompatibility study were performed. 

In general, a raise on cell proliferation at 24h and 48h was identified, except for the undiluted CERD at 48h. This data agrees with da Silva et al. ^10^ that related a reduction of pre-osteoblast cell growth after 48h in presence of calcium hydroxide paste associated with higher concentration of diclofenac sodium. Irrespectively of the dilution used, no difference was observed in the presence of Biodentine; however, cells submitted to higher dilution of MTA promoted a raise at 48h, corroborating with Takita et al.^23^ that showed the directly relation between of cell proliferation and calcium ion release and reported the cell proliferation increases in a dose-dependent manner after calcium chloride addition. By the way, previous data demonstrated a raise on cell metabolism in presence of MTA and Biodentine at initial time ^24^. On the other hand, CERD was able to impair the proliferation rate when compared with MTA at 48h. It has been described that NSAIDs suppressed proliferation and induces cell death of osteoblasts ^8^ ^,^ ^25^. In opposite, no cytotoxic at low concentration of diclofenac sodium were related by da Silva et al. ^10^. Thus, taken together, we believe that difference in relation to calcium ion release and presence of diclofenac sodium in CERD might explain our data. Therefore, it is clear that further investigations are required to better clarify the findings.

Tricalcium silicate-based cements are known to be biocompatible; however, initially, these products caused an intense inﬂammatory reaction that decreased by time ^3^ ^,^ ^4^ which also were observed in this study for all cements. In addition, no evidence of that the addition of NSAIDs interfere/modify the inflammatory response were identified. These findings can be support by Silva et al. ^10^ that verified that paste associated with diclofenac induced lesser inflammatory tissue reaction than when compared with pure calcium hydroxide paste by day 30, showing that the addition contributed to reduction of the inflammatory process.

Even with studies showing that NSAIDs suppress bone repair, growth, and remodeling *in vivo* ^25^, mineralization areas were identified in presence of CERD revealing that this experimental cement did not inhibit the calcium release and mineralization process. Thus, it is possible that the concentration of diclofenac sodium used was not able to alters and/or interfere on both, calcium release or hydration reaction, which may justify our findings. Moreover, mineralization areas in presence of MTA and Biodentine has been already described ^16^. 

Although, based on this data, it was possible to concluded that the association of diclofenac sodium did not interfere in setting time, radiopacity, solubility and ionic dissociation of CERD; moreover, it was able to impair the proliferation rate when compared to MTA at 48h. On the other hand, inflammatory response and mineralization ability also was not modified by diclofenac sodium. Besides, CERD demonstrated similar proprieties to others endodontic repair materials available.

## References

[1] Jitaru S, Hodisan I, Timis L, Lucian A, Bud M (2016). The use of bioceramics in endodontics - literature
review. Clujul Med.

[2] Camilleri J, Sorrentino F, Damidot D (2013). Investigation of the hydration and bioactivity of radiopacified
tricalcium silicate cement, Biodentine and MTA Angelus. Dent Mater.

[3] da Fonseca TS, da Silva GF, Tanomaru-Filho M, Sasso-Cerri E, Guerreiro-Tanomaru JM, Cerri PS (2016). In vivo evaluation of the inflammatory response and IL-6
immunoexpression promoted by Biodentine and MTA Angelus. Int Endod J.

[4] da Fonseca TS, Silva GF, Guerreiro-Tanomaru JM, Delfino MM, Sasso-Cerri E, Tanomaru-Filho M, Cerri PS (2019). Biodentine and MTA modulate immunoinflammatory response favoring
bone formation in sealing of furcation perforations in rat
molars. Clin Oral Investig.

[5] Bhavana V, Chaitanya KP, Gandi P, Patil J, Dola B, Reddy RB (2015). Evaluation of antibacterial and antifungal activity of new
calcium-based cement (Biodentine) compared to MTA and glass ionomer
cement. J Conserv Dent.

[6] Gan TJ (2010). Diclofenac: an update on its mechanism of action and safety
profile. Curr Med Res Opin.

[7] Díaz-Rodríguez L, García-Martínez O, Morales MA, Rodríguez-Pérez L, Rubio-Ruiz B, Ruiz C (2012). Effects of indomethacin, nimesulide, and diclofenac on human
MG-63 osteosarcoma cell line. Biol Res Nurs.

[8] Jenarthanan S, Subbarao C (2018). Comparative evaluation of the efficacy of diclofenac sodium
administered using different delivery routes in the management of endodontic
pain: A randomized controlled clinical trial. J Conserv Dent.

[9] de Freitas RP, Greatti VR, Alcalde MP, Cavenago BC, Vivan RR, Duarte MA, Weckwerth AC, Weckwerth PH (2017). Effect of the Association of Nonsteroidal Anti-inflammatory and
Antibiotic Drugs on Antibiofilm Activity and pH of Calcium Hydroxide
Pastes. J Endod.

[10] da Silva GF, Cesário F, Garcia AMR, Weckwerth PH, Duarte MAH, de Oliveira RC, Vivan RR (2020). Effect of association of non-steroidal anti-inflammatory and
antibiotic agents with calcium hydroxide pastes on their cytotoxicity and
biocompatibility. Clin Oral Investig.

[11] Ruiz-Linares M, Solana C, Baca P, Arias-Moliz MT, Ferrer-Luque CM (2021). Antibiofilm potential over time of a tricalcium silicate material
and its association with sodium diclofenac. Clin Oral Investig.

[12] Leão C, Borges A, Simões M (2020). NSAIDs as a Drug Repurposing Strategy for Biofilm
Control. Antibiotics (Basel).

[13] Cavenago BC, Pereira TC, Duarte MA, Ordinola-Zapata R, Marciano MA, Bramante CM, Bernardineli N (2014). Influence of powder-to-water ratio on radiopacity, setting time,
pH, calcium ion release and a micro-CT volumetric solubility of white
mineral trioxide aggregate. Int Endod J.

[14] Húngaro Duarte MA, de Oliveira El Kadre GD, Vivan RR, Guerreiro Tanomaru JM, Tanomaru-Filho M, de Moraes IG (2009). Radiopacity of portland cement associated with different
radiopacifying agents. J Endod.

[15] Cavenago BC, Del Carpio-Perochena AE, Ordinola-Zapata R, Estrela C, Garlet GP, Tanomaru-Filho M (2017). Effect of Using Different Vehicles on the Physicochemical,
Antimicrobial, and Biological Properties of White Mineral Trioxide
Aggregate. J Endod.

[16] Quintana RM, Jardine AP, Grechi TR, Grazziotin-Soares R, Ardenghi DM, Scarparo RK, Grecca FS, Kopper PMP (2019). Bone tissue reaction, setting time, solubility, and pH of root
repair materials. Clin Oral Investig.

[17] Duarte MA, Alves de Aguiar K, Zeferino MA, Vivan RR, Ordinola-Zapata R, Tanomaru-Filho M, Weckwerth PH, Kuga MC (2012). Evaluation of the propylene glycol association on some physical
and chemical properties of mineral trioxide aggregate. Int Endod J.

[18] Natu VP, Dubey N, Loke GC, Tan TS, Ng WH, Yong CW, Cao T, Rosa V (2015). Bioactivity, physical and chemical properties of MTA mixed with
propylene glycol. J Appl Oral Sci.

[19] Marciano MA, Guimarães BM, Amoroso-Silva P, Camilleri J, Hungaro Duarte MA (2016). Physical and Chemical Properties and Subcutaneous Implantation of
Mineral Trioxide Aggregate Mixed with Propylene Glycol. J Endod.

[20] Marciano MA, Duarte MA, Camilleri J (2016). Calcium silicate-based sealers: Assessment of physicochemical
properties, porosity and hydration. Dent Mater.

[21] Antunes Bortoluzzi E, Juárez Broon N, Antonio Hungaro Duarte M, de Oliveira Demarchi AC, Monteiro Bramante C (2006). The use of a setting accelerator and its effect on pH and calcium
ion release of mineral trioxide aggregate and white Portland
cement. J Endod.

[22] Ballal NV, Shavi GV, Kumar R, Kundabala M, Bhat KS (2010). In vitro sustained release of calcium ions and pH maintenance
from different vehicles containing calcium hydroxide. J Endod.

[23] Takita T, Hayashi M, Takeichi O, Ogiso B, Suzuki N, Otsuka K, Ito K (2006). Effect of mineral trioxide aggregate on proliferation of cultured
human dental pulp cells. Int Endod J.

[24] Araújo LB, Cosme-Silva L, Fernandes AP, Oliveira TM, Cavalcanti BDN, Gomes JE, Sakai VT (2018). Effects of mineral trioxide aggregate, BiodentineTM and calcium
hydroxide on viability, proliferation, migration and differentiation of stem
cells from human exfoliated deciduous teeth. J Appl Oral Sci.

[25] Chang JK, Wang GJ, Tsai ST, Ho ML (2005). Nonsteroidal anti-inflammatory drug effects on osteoblastic cell
cycle, cytotoxicity, and cell death. Connect Tissue Res.

